# Orbitrap journey: taming the ion rings

**DOI:** 10.1038/s41467-019-11748-y

**Published:** 2019-09-09

**Authors:** Alexander Makarov

**Affiliations:** Thermo Fisher Scientific, Hanna-Kunath-Str. 11, 28199 Bremen, Germany

**Keywords:** Proteomics, Mass spectrometry, Analytical chemistry, Techniques and instrumentation

## Abstract

The establishment of the Orbitrap analyzer as a major player in mass spectrometry based proteomics is traced back to the first public presentation of this technology 20 years ago; when a proof-of-principle application led the way to further advancements and biological applications.

## A physicist leaves his comfort zone

Any jubilee celebration is a great opportunity to recall events that looked insignificant at the time but, like a good wine, gained appreciation over the course of decades. My first public presentation of Orbitrap technology at the annual conference of American Society for Mass Spectrometry (ASMS) in Dallas, TX, 20 years ago was lucky enough to land into this category as witnessed by an invitation to contribute this article.

Looking back, this talk turned out to be quite a stressful experience. The microphone failed and my slides were warping in the hot slide projector because they had picked up moisture in the humid Texan summer on my walk from a cheap hotel. So the talk became a test of character—but this was not the first or the last time when Orbitrap forced me to step out of my comfort zone.

The Orbitrap journey started many years before, probably during my diploma when I tried to implement a little-known type of electrostatic field^1^ for ion separation on the time of flight (TOF) principle. This field later formed the basis of the Orbitrap analyzer, but initial TOF results were not that good, albeit still contributing to my PhD thesis on ion optics. Even though I was focused on completing my thesis, I could not fail to notice a thorough economic and political meltdown of the country around me. This eventually forced me to look for scientific opportunities beyond Russia. A short stint of consulting at a mass spectrometry company in Manchester, UK, in 1993 and a post-doc position at Warwick University in 1994–1996 provided me with a good circle of life-long acquaintances and friendships that defined subsequent events.

Importantly, I became witness to a revolution in ionization that enabled the analysis of fragile peptides and proteins. This revolution had started just a few years before with the introduction of electrospray by John Fenn^2^ and Lidia Gall^3^ and matrix assisted laser desorption/ionization (MALDI) by Koichi Tanaka^4^, Franz Hillenkamp and Michael Karas^5^. It was not easy for a physicist to understand biological applications - but excitement in the academic world made it quite clear that exactly these applications were going to drive instrumentation advances over the next decades.

When my new friends formed a company called HD Technologies and I joined them in 1996 in Manchester, UK, our long-term dream was to move the needle in those biological applications of mass spectrometry. One of the company’s founders, Steve Davis, emphasized that we need to develop something really remarkable to get out of the cellar where the company started. Once he joked about a mass spectrometer that combines the ultra-high resolution and mass accuracy of a Fourier transform ion cyclotron resonance (FT-ICR) analyzer with the sensitivity of a linear TOF instrument, all this packaged within a small box typical of an ion trap or a single quadrupole. Funnily enough, this is approximately where we eventually arrived after all these years!

## Perfect electrodes welcome a rapid fire of ions

Meanwhile, the company had to survive by picking any job, however small or large. These projects usually dealt with the development of compact TOF analyzers for different ion sources: electron impact, MALDI, etc. The hunter-gatherer unpredictability of finding next orders was continuously reminding us that we need to somehow get out of this perpetual cycle of survival.

These worries motivated me to use my weekends and holiday time to come up with an idea that could pave the way towards the desired ideal instrument. A distant encounter with FT-ICR at Warwick University and ion traps at an ASMS conference created a sense of intellectual romance with the idea of ion trapping while my long-standing dislike of large magnets and radio-frequency coils naturally guided me back to electrostatic fields. I already knew that moving charges can be held stable in such fields – just like a sputnik on an orbit around the Earth. However, I was also aware of many practical pitfalls associated with the electrostatic field^1^ used in my diploma, the most important one being not the trapping of ions but rather their injection into a stable orbit from outside the field.

Initially, I planned ion injection as an orderly symphony of multiple voltage ramps on numerous precise electrodes^6^. We used this patent application as a basis for a grant submission to the UK Department of Trade and Industry and our company became one of few winners. In spite of the modest scale of the £50,000 grant, it became a blessing for our four-people company as it solidified the entire endeavor both with legitimacy and deadlines.

It came as a cold shower to find out that no engineer could ever dare to realize such complexity in reality. In despair, I decided to cut this Gordian Knot by rapidly hammering ions into the trap not from a continuous but from a pulsed ion source. An old nitrogen laser became the basis for such a source and existence of a common starting time point for all ions made it possible to excite ion motion just by injecting them at an offset from the trap equator. Such injection required a small slot in the outer electrode that caused hideous non-linear scattering and distortion of ion motion. It took several years to get its geometry right.

Pulsed injection enabled a simpler geometry of electrodes, which I submitted to our machining subcontractor with a request to get the best shape accuracy possible. On a brand-new machine they made electrodes that worked nicely. Unknown to all of us, this happened to be a win in the lottery as nobody was able to repeat this success on any machine of that or similar type. Only many years later and only after herculean efforts and specialized development were our suppliers able to machine such electrodes with reasonable success rate.

As our mechanical engineer Robert Lawther and electronic engineer Andrew Hoffman were advancing through further building blocks of breadboard, our learning continued and especially intensified when real ions finally started flying. Naturally, a plethora of instabilities, noise signals and other attributes of sensitive image current detection were uncovered and addressed on the fly. With every such improvement, resolving power was increasing, reaching around 47,000 for sodium ions by the time of my first ASMS presentation and 150,000 by the time the consequent paper was submitted^7^. Mass accuracy of several parts per million with internal calibrant and a mass range beyond 1000 Da was also demonstrated by that time.

## A mass analyzer unfolds its potential

While modest by modern standards, this performance attracted attention of both academics and vendors. This resulted in lively discussions on possible fatal flaws of the Orbitrap technology that would prevent it from entering mainstream mass spectrometry. Although disheartening at the time, these discussions helped to formulate a double-digit number of further directions of work for years ahead.

As it turned out, the first presentation of the Orbitrap marked completion of probably just one per cent of the journey to the first commercial instrument, though I was blessed enough not to know this truth at that time. However, it did help with the acquisition of HD Technologies by Thermo Quest Inc., which in turn contributed to the formation of a company known now as Thermo Fisher Scientific. This journey deserves its own story^8^ but it reached its culminating point with the launch of the LTQ Orbitrap instrument in 2005. The instrument was enthusiastically received by the scientific community. However, this success was actually built upon the hard-earned ease-of-use of previous proteomics workhorses such as the linear trap/FT-ICR hybrid mass spectrometer. The Orbitrap detector just made mass accuracy and high resolution as sensitive, robust and easy to achieve as nominal mass detection before. This became the major game changer for biomolecular mass spectrometry—first in the area of mass spectrometry-based proteomics and later in the fight against doping and the structural analysis of small molecules.

Second generation benchtop quadrupole/Orbitrap hybrid instruments of the Q Exactive family democratized the Orbitrap feature set, making it compatible with a broader range of metabolomics, screening, biopharma and other applications. The third generation tribrid mass spectrometers, such as Orbitrap Fusion, Lumos and now Eclipse, added an increasingly rich variety of fragmentation methods and intelligent on-the-fly decision making to the proteomics toolset. This legacy continues with the fourth generation of hybrid instruments of the Exploris family. Like Industry 4.0, they follow the vision of seeming simplicity of operation that hides extensive networking, self-diagnostics and decentralized decision-making.

With many thousands of instruments working 24/7 around the globe, Orbitrap capabilities have been expanding enormously over these years in multiple dimensions: from detecting elements and isotope ratio analysis to protein complexes and intact viruses; from integrating liquid separations to gas chromatography to in-surgery diagnostic tools and ambient imaging; from disease pathway elucidation to biopharmaceutical quality control. As Orbitrap instrumentation continues to develop at a rapid pace, we may expect that further surprising dimensions will continue to open for all of us who strive towards a safer, cleaner and healthier world.
